# Seasonal Variations of Fine Root Dynamics in Rubber-*Flemingia macrophylla* Intercropping System in Southwestern China

**DOI:** 10.3390/plants11202682

**Published:** 2022-10-12

**Authors:** Farkhanda Bibi, Durairaj Balasubramanian, Muhammad Ilyas, Jan Sher, Hamz Ali Samoon, Muhammad Hayder Bin Khalid, Hesham F. Alharby, Ali Majrashi, Sameera A. Alghamdi, Khalid Rehman Hakeem, Muddaser Shah, Shabir A. Rather

**Affiliations:** 1 CAS Key Laboratory of Tropical Plant Resources and Sustainable Use, Xishuangbanna Tropical Botanical Garden Chinese Academy of Sciences, Mengla 666303, China; 2 Department of Botany, Abdul Wali Khan University Mardan, Mardan 23200, Pakistan; 3 Department of Botany, Arunachal University of Studies, NH-52, Namsai 792103, Arunachal Pradesh, India; 4 CAS Key Laboratory of Tropical Forest Ecology, Xishuangbanna Tropical Botanical Garden, Chinese Academy of Sciences, Mengla 666303, China; 5 Center for Integrative Conservation, Xishuangbanna Tropical Botanical Garden, Chinese Academy of Sciences, Mengla 666303, China; 6 Principal Scientific Officer Pakistan Agricultural Research Council-Water and Agricultural Waste Management Institute, Tando Jam 70050, Pakistan; 7 National Research Center of Intercropping, The Islamia University of Bahawalpur, Bahawalpur 63100, Pakistan; 8 Department of Biological Sciences, Faculty of Science, King Abdulaziz University, Jeddah 21589, Saudi Arabia; 9 Department of Biology, College of Science, Taif University, P.O. Box 11099, Taif 21944, Saudi Arabia; 10 Princess Dr. Najla Bint Saud Al-Saud Center for Excellence Research in Biotechnology, King Abdulaziz University, Jeddah 21589, Saudi Arabia; 11 Department of Public Health, Daffodil International University, Dhaka 1341, Bangladesh

**Keywords:** fine root biomass, monocultures, intercropping, rubber, *Flemingia macrophylla*, soil nutrients (N, P, K)

## Abstract

Intercropping cover crops with trees enhance land productivity and improves the soil’s physio-chemical properties while reducing the negative environmental impact. However, there is a lack of quantitative information on the relationships between fine root biomass and available soil nutrients, e.g., nitrogen (N), phosphorus (P), and potassium (K), especially in the rubber-*Flemingia macrophylla* intercropping system. Therefore, this study was initiated to explore the seasonal variation in fine root biomass and available soil nutrients at different stand ages (12, 15, and 24 years) and management systems, i.e., rubber monoculture (mono) and rubber-*Flemingia macrophylla* intercropping. In this study, we sampled 900 soil cores over five seasonal intervals, representing one year of biomass. The results showed that the total fine root biomass was greater in 12-year-old rubber monoculture; the same trend was observed in soil nutrients P and K. Furthermore, total fine root biomass had a significant positive correlation with available N (*p* < 0.001) in rubber monoculture and intercropping systems. Thus, it suggests that fine root growth and accumulation is a function of available soil nutrients. Our results indicate that fine root biomass and soil nutrients (P and K) may be determined by the functional characteristics of dominant tree species rather than collective mixed-species intercropping and are closely linked to forest stand type, topographic and edaphic factors. However, further investigations are needed to understand interspecific and complementary interactions between intercrop species under the rubber-*Flemingia macrophylla* intercropping system.

## 1. Introduction

Extensive deforestation has occurred in many parts of the world to support rubber monoculture to meet the demand [1]. In Xishuangbanna, China, large-scale rubber plantation has significantly increased the depletion of soil carbon (>50%) [2]: causing soil acidification: changes in the composition and quantity of nutrients, forest degradation, and reduced vegetation biomass [3]. Such changes in above-ground vegetation will significantly affect the below-ground properties and soil biodiversity. Fine root systems have studied the competition between soil organisms [4]. Therefore, intercropping is seen as a sustainable farm management practice.

In rubber plantations of Xishuangbanna and Southeast Asia, intercropping ensures resource sustainability [5]. Amongst roots of plants, fine roots (≤2 mm, diameter) are essential as they play a crucial role in soil nutrient dynamics [6]. Hence, understanding whether intercropping can conserve soil resources and enhance productivity and ecosystem services is important. Hence, studying differences in the growth and production of fine root dynamics of rubber monoculture and rubber intercropping can help elucidate their role in soil nutrient biogeochemistry and overall ecosystem functions and services. Fine roots characterized by relatively short lifespan (a few months), faster growth, and rapid turnover and contribute 18 to 58% of total nitrogen to forest soil [7,8]. Despite representing only, a small percentage of plant biomass, fine roots utilize about a third of annual global net primary productivity (NPP). Fine roots are a major source of soil carbon through root decomposition and rhizodeposition [9,10]. More than 50% of the annual NPP is allocated to roots in many forests as they decompose relatively faster than the above-ground plant parts [11,12]. Although fine roots represent only 5% of total tree biomass, they consume between 30 to 50% of annual gross primary production (GPP) [13]. As a part of below-ground processes, fine roots play a crucial role in tree growth, stand development, and the biogeochemical cycling of nutrients, primarily carbon (C), nitrogen (N), and water in forest ecosystems [14]. Fine roots are the drivers of a significant C and nutrient pool and are closely associated with the soil, thus contributing to the formation of soil organic matter (SOM) [15]. Globally, fine roots contribute about 33% of the NPP and account for 3–30% of the total root biomass in terrestrial ecosystems. Consequently, this leads to competition amongst species with a substantial effect on the below-ground competition via fine roots [16].

Many plant species such as pineapple, ginger (*Zingiber officinale*), turmeric (*Curcuma longa*), and tea (*Camellia sinensis*) are widely cultivated as intercrops in rubber-based agroforestry systems [17,18]. Intercropping with nitrogen-fixing species (NFS) [19,20,21] can enrich the soil quality and productivity of rubber plantations [22]. The present study focused on the intercropping legume *Flemingia macrophylla* (Willd.) Kuntze ex Merr. as it is an excellent NFS cover crop with dense growth, low decomposition rate and accumulation of SOM, capability to resist occasional flooding, and moderate drought tolerance. A previous study also showed that intercropping of *F. macrophylla* in rubber-based agroforestry systems enhanced soil C and N storage and conserved water [23]. Rubber-*F. macrophylla* intercropping systems have become widespread in Southwestern China [24]. However, so far, no studies have been attempted to understand how intercropping of *F. macrophylla* can change fine root biomass dynamics in different aged rubber plantations and how rubber-*F. macrophylla* systems impact nutrient cycling in rubber plantations. Hence, assessment of fine root dynamics and nutrient status of rubber-*F. macrophylla* intercropping can benefit in understanding the below-ground competition and how the species diversity (monoculture rubber plantation Vs intercropping) affects below-ground nutrient (C and N) storage.

Though fine root biomass is crucial in C and N cycling in plantation systems, growth and biomass production is significantly affected by the plantation stand age. Several studies have demonstrated an increasing trend in soil N concentrations with stand age [25]. However, other studies of secondary forest ecosystems have found that C sequestration decreases with the stand age [26,27,28], suggesting that some forests and plantations might become less productive over time, possibly due to the depletion of soil nutrients [29,30]. Therefore, it is unclear how the stand age affects the fine root biomass and whether there is an interaction between different stand ages and soil nutrients across different soil depths. Further, it is unclear how *F. macrophylla* intercropping alters spatiotemporal dynamics of fine root biomass in a rubber plantation. Hence, in the present study, we attempted to explore the seasonal variations in fine root biomass dynamics and nutrient stoichiometry across different stand ages in rubber monoculture and rubber-intercropping systems. The main objectives of this study were (i) to examine the influence of the intercropping of *F. macrophylla* on different-aged (12, 15, and 24 years) rubber monoculture plantations on fine root biomass rates at 0–60 cm soil depth (ii) to evaluate the seasonal variations in fine root biomass and soil available soil nutrients (C, N, P, and K) to know the relationship between fine root biomass and nutrients in different stand age development. It was hypothesized that rubber intercropping systems would accumulate more fine roots than rubber monoculture. We also hypothesized that the seasonal changes in monoculture and intercropping systems would influence available soil nutrients.

## 2. Results

### 2.1. Seasonal Dynamics of Fine Root Biomass

**Figure 1:**
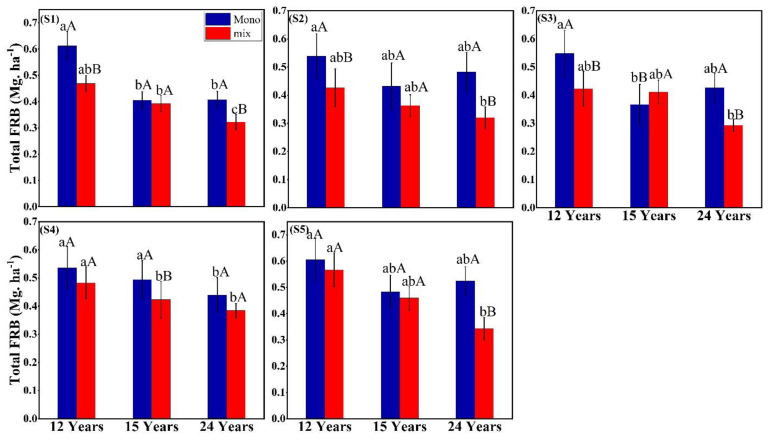
Total fine root biomass under different stand ages (12, 15, and 24 years) and seasonal intervals ((S1), March 2018; (S2), June 2018; (S3), September 2018; (S4), December 2018 and (S5), March 2019) in the two management systems, i.e., rubber monoculture (mono) and rubber-*F. macrophylla* intercropping (mix). The uppercase letters represent a significant difference between the two management systems, and the lowercase letters represent the significant difference between the three stand ages at *p* < 0.05 (mean ± SE, n = 3).

Cumulative fine root biomass and its distribution pattern were estimated at stand level with five samplings times (one year from March 2018–2019) covering three seasons (dry, rainy, and foggy cool). Fine root biomass was strongly affected by season and soil depth (*p* < 0.001) based on the mixed-effect model (Table 1). The linear mixed effect model showed no interaction between the cropping systems (monoculture Vs. intercropping). Meanwhile, the Post-hoc Tukey test showed a significant difference between stand ages at each seasonal interval and between two cropping systems (Figure 1). It was found that seasons × soil depth and stand age × soil depths had a significant (*p* < 0.001) effect on fine root biomass. The fine root biomass exhibited a contrasting result in the intercropping systems, i.e., intercropping systems had lower fine root biomass than rubber monoculture systems, except under 15-year-old intercropping systems during S3 (Figure 1). Fine root biomass negatively correlated with the increasing stand age; the young age (12 years) plantation contributed to greater fine root biomass than the 15- and 24-years old stands. However, fine root biomass significantly increased during S1 and S5 monoculture systems (Figure 1). Similarly, the intercropping system’s highest fine root biomass was observed in 12 years during S5. The relationship of fixed effect in the mixed model explained the 0.76 (marginal R^2^) of the total variances, and the random effects explained 0.82 of the total variances (conditional R^2^) (Table 1).

### 2.2. Total Fine Root Biomass Contribution of Different Species

The highest fine root biomass of *F. macrophylla* was found in the 15 years old stand during S2. Fine root biomass and dead fine root necromass were lower in 15 years old intercropping systems during S4 (Table 2). Further, the intercropping of *F. macrophylla* in rubber monoculture changed the spatial distribution of living fine rubber roots; however, fine root biomass is strongly controlled by the stand age of rubber plantation.

### 2.3. Seasonal Dynamics of Available Soil Nutrients

#### 2.3.1. Available Soil Nitrogen (N)

Soil N concentration was significantly affected by season (*p* < 0.001) (Table 1). However, there was no significant (*p* < 0.695) difference between cropping systems. N concentration was significantly affected by the interaction of seasons and cropping systems and seasons and stand age (Table 1). There was a significant interaction between seasons × cropping systems × stand age (*p* < 0.001). The N contents were significantly higher during S1 and decreased during S2 and S4 (Table 3). The overall N was higher in the intercropping system than in monoculture. Hence, soil N was significantly higher in a matured intercropping system (24 years). The relationship of fixed effect in the mixed model explained about 0.78 (marginal R^2^) of the total variances, and the random effects explained about 0.79 of the total variances (conditional R^2^) (Table 1).

#### 2.3.2. Available Soil Phosphorus (P)

The linear model results showed that soil P concentrations were strongly affected by seasons, cropping systems, and stand ages (*p* < 0.001). In addition, P concentration was affected by the interaction of season and cropping systems, season interaction with age, and management interaction with age (*p* < 0.001). P concentrations were significantly higher in young stand age rubber monoculture than in the young stand age intercropping system. Similarly, P concentrations significantly varied across cropping systems of different stand ages (Table 3). The soil P concentration was higher during S4 in mature stand age (24 years) of rubber monoculture. The relationship of fixed effect in the mixed model explained the 0.85 (marginal R^2^) and random effects of 0.87 (conditional R^2^) of the total variances (Table 1).

#### 2.3.3. Soil Available Potassium (K)

Soil available K concentration was significantly affected by all the fixed factors, season, treatments, age, and soil depths (*p* < 0.001). Soil K concentration was significantly (*p* < 0.001) affected by seasons and their interaction with management, season interaction with age, and management interaction with age. Further, there was a significant interaction between season × management × age (*p* < 0.001) (Table 1). Soil K concentrations were significantly higher in rubber monoculture than in the intercropping systems. Soil K was significantly higher under young age (12 years) in rubber monoculture. Similarly, seasons strongly influenced the soil K concentration under all the stand ages. Greater K concentration was recorded under 12 years old rubber monoculture during S3 and lower during S4 under 24 years old intercropping systems (Table 3). The relationship of fixed effect in the mixed model explained the 0.64 (marginal R^2^) and the random effects of 0.77 (conditional R^2^) of the total variances (Table 1).

### 2.4. Effect of Available Soil Nutrients on Fine Root Biomass

**Figure 2:**
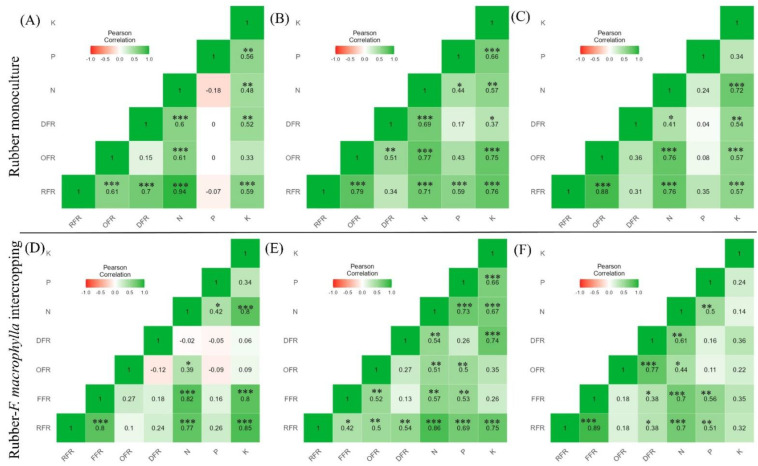
Correlational matrix of total fine root biomass with different species in rubber monoculture ((**A**) = Correlogram of 12 years, (**B**) = Correlogram of 15 years, and (**C**) = Correlogram of 24 years) and rubber-*F. macrophylla* intercropping agroforestry system ((**D**) = Correlogram of 12 years, (**E**) = Correlogram of 15 years, and (**F**) = Correlogram of 24 years). The acronyms represent fine roots of rubber (RFR), *F. macrophylla* (FFR), other (non-tress) (OFR), dead (DFR), and available nutrients (N, P, and K). The colored legend gradients represent correlation coefficients of r values from dark green (+1.0) to light red (−1.0). All coefficients were analyzed through the Pearson correlation at significance level: ***, *p* < 0.001; **, *p* < 0.01, *, *p* < 0.05 for the possible pair variables in the matrix.

Overall, fine root biomass had a significant correlation (*p* < 0.05) with other species (Figure 2). In 12 years, old stands of rubber monoculture, species-wise fine root biomass had a significant positive correlation with the available nutrients (except for P). In addition, RFR, OFR, and DFR had a strong positive correlation with soil N. Further, under 12 years old stands of intercropping systems, RFR significantly (r = 0.8) correlated with FFR. In 15 years stands of rubber monoculture, RFR positively correlated with all variables except DFR. At 24 years old stands of rubber monoculture, RFR had a positive correlation with OFR (r = 0.9), N (r = 0.8), and K (r = 0.5). Similarly, OFR and DFR have a significant correlation with N and K. Interestingly, after 24 years of intercropping systems, all variables had a significant positive correlation with available nutrients (except for K).

## 3. Discussion

Most of the previous studies on soils of tropical forests focused on the upper soil layers (0–30 cm). In the present study, we investigated the seasonal variations in fine root biomass and soil available nutrients (N, P, K) concentration across 0–60 cm soil depth of different stand ages in the two cropping systems, i.e., rubber monoculture and rubber-*F. macrophylla* intercropping system.

### 3.1. Fine Root Biomass and Nutrients Dynamics in Two Management Systems

The rubber-*F. macrophylla* intercropping system did not affect the total fine root biomass. Hence, there is no direct evidence of tree species diversity and intercropping system’s effects on fine root biomass under the rubber-*F. macrophylla* system, thus rejecting our first hypothesis. Meinen et al. [31] also found no significant difference in fine root biomass among different tree species diversity systems (one, three, or five dominant tree species). However, Domisch et al. [32] reported that intercropping of deciduous species in monoculture plantations produced high fine root biomass in southern Finland. Similarly, Jacob et al. [33] found a positive effect on fine root biomass with *Castanea dentata* introduced to the forest. As an indicator of the total soil space-filling of fine roots in the monoculture systems, fine root biomass heterogeneity contained higher above-ground biomass than the mixed-species stands [34]. Dybzinski et al. [35] also described that fine root biomass increases with species diversity compared to a monoculture plantation. Thus, we conclude that less fine root biomass in mixed forests in our study could be explained by more effective soil resource uptake per unit of fine root biomass under intercropping system over the previous years; for example, *F. macrophylla* was planted in 2010, eight years before our samplings.

In the present study, the effect of competition for N under the intercropping system was not clear. In contrast, soil P and K concentrations differed significantly between the cropping systems. Due to the high mobility of N compounds within the soil, P and K are leached easily through preferential flows during the rainy season [36]. The significant increase in the P and K concentrations in the rubber monoculture (Table 3) suggests enhanced nutrient acquisition by the rubber roots biomass. However, the low soil P and K concentration in the rubber-*F. macrophylla* intercropping implies that intense interspecific competition may result in less P and K input or output within the soil.

The effects of competition for nutrients are weakly reflected in the soil P concentration because of its low mobility [37]. The rubber trees had a significant advantage in competing for soil P because their roots were more developed than the other intercrops. Similarly, K was substantially higher under the young stand age of rubber monoculture (Table 3), which depends on the type of clay content (mainly smectites) in soil and the level of leaching corresponding with other studies [38]. The previous research demonstrated that multi-cropping agroecosystems showed increased soil organic matter and C and N contents compared to monoculture [39]. However, we found rubber monoculture exhibited the highest soil total C, available P, and K concentrations across the soil profile, whereas C and N loss under rubber-*F. macrophylla* intercropping could be due to a higher decomposition rate and leaching. Moreover, *F. macrophylla* is an N-fixing legume, has a function that has very high requirements for P and K [40], and consequently may have depleted through extraction in the earlier years, causing the lower P and K content in the intercropping stands than the monoculture (Table 3). It is supported by the negative correlation between fine root biomass and available P (Figure 2). 

### 3.2. Effect of Stand Ages

Stand age significantly affected the total fine root biomass and nutrients (except N) under young stands (12 years old) of rubber monoculture and rubber-*F. macrophylla* intercropping system compared to the mature stands (15 and 24 years old). Similarly, Wang’s [41] findings supported our results that the increased fine root biomass under rubber monoculture decreases with stand ages. In contrast, young rubber plantation stands use more water than mature plantation stands, thus producing higher fine root biomass. Pei et al. [42] consistently found that fine root biomass increased within the age of 7-year-old and 17-year-old stands and then decreased in the 25-year-old Chinese Fir plantation. In contrast, Valverde-Barrantes et al. [43] found that fine root biomass increased with stand age in high species evenness in a mixture of plantations dominated by two boreal tree species. In the present study, higher fine root biomass under young stand age (Figure 1) can be attributed to increasing N and altered species composition and biomass across stand age [25,44]. A more significant proportion of fine roots in the young stand is an efficient strategy to enhance the absorption of available nutrients by exploring large soil volumes and enabling stand recovery [45,46]. The significant effect of stand age and seasons suggest that environmental variability between different ages and seasons creates distinct effects on the soil N, P, and K concentrations. Soil N contents did not differ significantly between cropping systems. However, N contents significantly varied between stand ages, i.e., higher N under the mature stands than in young stands (Table 3). The previous inconsistent study [47] also found high N concentration under mature stand (20 years old) of *Gliricidia*-based intercropping systems.

Consequently, lower fine root biomass is needed for nutrient uptake in mature age than in young age, and the above-ground/below-ground biomass ratio decreases with tree age [48]. On the other hand, decreasing P and K concentrations under mature stands of intercropping systems could be attributed to a high amount of P loss from consecutive latex tapping of mature rubber trees along with soil runoff and erosions over time. Soil available K is mostly easily leached by runoff; hence, K’s accessible by fine roots is lower than the N or P [49,50]. These results suggest that total P and K gradually deplete with plantation stand age. Thus, P and K fertilizers might be necessary to maintain rubber latex productivity under mature rubber stands [29]. Generally, the reason for lower fine root biomass under mature rubber stands could be the lower soil fertility (available P and K), and rubber trees may compromise the capability to sustain biomass during the dry season. We found a clear declining trend in the soil available P and K with the increasing stand age; however, we did not find any apparent trends in available N across stand ages. In contrast, previous studies observed increasing soil N concentrations with the increase in stand age [46,51].

The fine root biomass was greater during the dry season (Figure 1), although peaking was expected during the rainy season when rubber trees were physiologically active in capturing nutrients [52]. This contrasts with many other previous observations from northern savannah, Australia [53], California, USA [54], and boreal forest [8] where the maximum biomass was found during the rainy season. Our result is similar to temperate forest studies, wherein standing root biomass peaked during the dry season when roots grew throughout the winter in the absence of leaves when the trees are physiologically inactive [55]. The maximum fine root biomass was observed during the dry season in some tropical forests, such as central Sulawesi, Indonesia, and the eastern Amazon, Brazil [56]. Rubber-*F. macrophylla* intercropping systems will be enormously beneficial under N-limited soil like rubber-grown soil, reducing the need for N fertilizers. This is consistent with the study of Malhotra et al. [57], where soil N contents were high in the dry site and low in the wet site, suggesting that N varies with site conditions, including soil types. Furthermore, we found higher P concentrations during the rainy season. In contrast with our results, the soil nutrients (total C, N, P, K, and Ca) during the dry season were higher than in other sampling times in intercropping systems [58]. In rubber growing areas of Xishuangbanna, soils are generally deficient and inactive. Soil available P exhibited a marked seasonal variation, reflected in different cropping systems and plantation stand ages [40]. This seasonal variation in soil nutrients appeared due to plant phenology, which influences the nutrient cycle by mediating plant nutrient uptake, litterfall, and soil biodiversity. Overall, the seasonal agronomic activities (e.g., rubber tapping) also substantially affect spatiotemporally soil nutrients.

### 3.3. Response of Fine Roots Biomass towards Soil Available Nutrients

In correspondence with other findings, available soil N was positively correlated with fine root biomass [59,60]. This finding suggests that N foraging is ubiquitous regardless of cropping systems and stand age and indicates that higher fine root productivity in intercropping systems benefited partly from the N foraging behavior [61]. However, in contrast with our study, Xu et al. [62] showed a weak or no correlation between available soil N and fine root biomass. They explained that higher soil N availability causes fine root mortality and declines in fine root biomass [63,64]. In contrast, P limits the primary production of fine roots, which explains the linear increase in fine root necromass with P availability [65]. Gower [66] found a negative relationship between fine root biomass and soil P availability. In contrast, there was a positive relationship between soil P concentration and fine root biomass, suggesting that higher P concentration on the forest floor controls fine root biomass production. Soil available K is easily leached by runoff; hence, K accessibility by fine roots is often limited compared to N or P [38]. Previous studies demonstrated that fine root production increased with the availability of soil N and P [67,68]. Thus, the variation in fine root biomass with soil nutrients indicated that root systems are highly resilient in response to environmental factors and can change their production at the stand level.

## 4. Materials and Methods

### 4.1. Study Site

The experiment was carried out in Xishuangbanna Dai Autonomous Prefecture, Yunnan province, Southwest China, (21°33′ N, 101°28′ E). The climate is tropical monsoon type, with a dry season between November to April, a rainy season from May to October, and a foggy, cool season from November to February with little rain but heavy fog in the morning and evening, which could compensate for insufficient water derived from rain. In the past 50 years, the annual average temperature has been around 21.5 °C, with a maximum monthly mean temperature of 26.5 °C in June and a minimum monthly mean temperature of 17.1 °C in December. The annual mean rainfall was 1500 mm, and more than 85% in the rainy season [69]. The annual average sunshine was 1850 h, with a high atmospheric humidity of 86% [70]. The soils are classified as oxisols, approximately 2 m deep overlying arenaceous shale sediments [71]. The native vegetation represents tropical seasonal rainforest, fragmented into small patches because of rubber tree (*Hevea brasiliensis*) plantations and construction [72].

### 4.2. Collection of Fine Roots Samples

**Figure 3:**
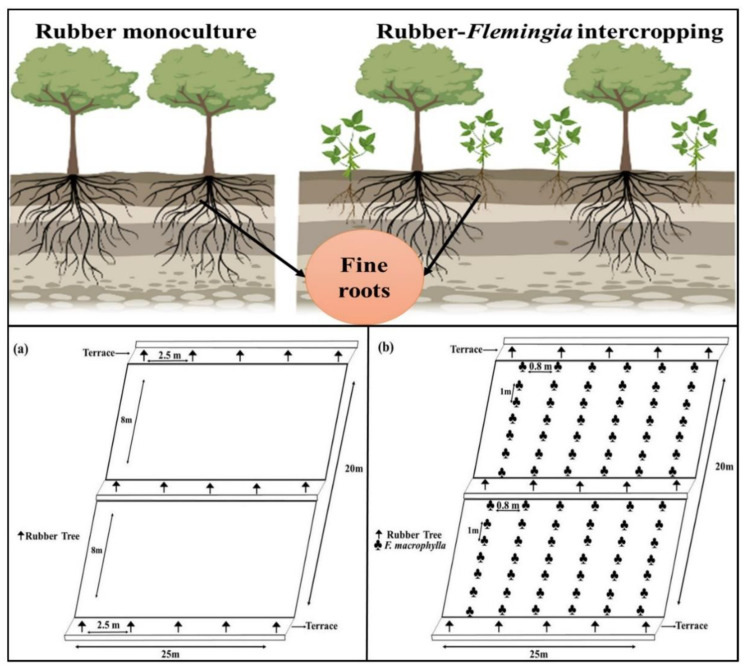
Schematic layout of plants in the specification in (**a**) rubber monoculture and (**b**) rubber-*F. macrophylla* intercropping.

The study site was dominated by rubber monoculture planted in 1994, 2006, and 2003 with a conventional 2.5 m × 8 m spacing arrangement (Figure 3). In June 2010, seven lines of the native *F. macrophylla* species, well adapted to the tropical climate and local soil conditions, were interplanted in a section of each monoculture stand at a density of 0.8 m × 1.0 m inter-row spacing with no further agronomic practices [40]. We collected soil cores from three sample subplots (25 m × 20 m) in each of the six stands, namely 12, 15, and 24-years-old rubber monoculture and agroforestry (each age had two plantations mono and mix) in 1 hectare in March 2018. We extensively assessed samples for 3-month optimal intervals for fine root biomass, i.e., seasonal intervals (S1, March 2018; S2, June 2018; S3, September 2018; S4, December 2018, and S5, March 2019). S1, S4, and S5 represented the dry season, while S2 and S3 represented the rainy season. All sites were managed similarly, except *F. macrophylla*, which was clipped approximately 30 cm above the ground and subsequently covered at the end of each year. We extracted soil cores from 0–60 cm depth with the help of a soil drill hand-boring auger (9 cm diameter). We took 10 soil cores randomly in each subplot; hence for each stand age (mono/mix), 30 soil cores samples were collected, which provided a total of 900 soil core samples (6 subplots plots (3 mono/3 intercrops) × 10 soil core × 3 stand ages × 5 seasons), for the determination of fine root dynamics. The extracted soil cores were kept in double-sealed plastic bags with name tags for identification. For nutrient determination, we took one soil core from each subplot and collected 18 soil cores in each season from a total of five seasons; we used 90 soil core samples for determining nutrients to explore how they varied amongst seasons and stand types and ages. Soil samples were air dried and sieved (2 mm) for further physio-chemical analysis. The descriptions of stand characteristics are shown in Table 4.

### 4.3. Estimation of Fine Root Biomass

The soil core was soaked in the water for eight hours to remove the soil and coarse fragments and to separate fine roots from coarse roots. In the water column, the fine roots floated on the water surface, while coarse fragments and soil would settle on the bottom [73]. We used a 2 mm mesh size sieve to clean fine roots from soil residues. Roots > 2 mm were collected using tweezers and discarded. Fine roots of different trees ≤ 2 mm in diameter were collected. Fine roots were classified according to their physiological status, live vs. dead, and based on the root’s texture, shape, and color. Fine roots were categorized as live if they were pale, elastic, flexible with a whitish cortex, and free of decay [74]. Roots were categorized as dead if the color was black or brown, rigid and inflexible in different stages of decay, and with a dark-colored cortex [75]. Fine roots were divided into the following classes: (i) rubber FR (RFR), (ii) *F. macrophylla* (FFR), (iii) other FR (OFR) (herbs), and (iv) dead FR (DFR) using a combination of various morphological characteristics. These included: (i) color and odor—fine rubber roots were pale brown, moderately coarse but even, with an unpleasant odor; *F. macrophylla* roots were dark brown, odorless, the taste was pungent, and non-tree (other FR) roots were white or yellow (ii) size of other fine roots—non-tree FR was fine in comparison with other tree roots (iii) branching pattern and root hairs of FR—rubber FR were less branched than those of *F. macrophylla*, and other FR (non-tree roots), whereas other FR contained more small hairs than fine tree roots. We distinguished all fine root samples of different species based on these characteristics, using a stereomicroscope when needed. The sorted fine roots were kept under the sunlight for three days to remove the moisture content and then dried at 70 °C for 48 h and weighed using an electronic balance (maximum weight: 210 g) following which the fine root biomass (Mg·ha^−1^) was calculated using the following formula:

Fine root biomass (Mg·ha^−1^) = (root dry weight (g) × 100)/(π × 4.5^2^) (1)

### 4.4. Soil Nutrient (C, N, P, and K) Determination

The soil was dried at 105 °C to constant weight for at least 24 h, then sieved through a 0.2 mm mesh and kept in plastic bags for nutrient analysis. The total C and N were determined by an elemental analyzer (Vario MAX CN-Analyzer, manufacturer Elementar Analysensysteme GmBH, Langenselbold, Germany). To determine total P and K content, acid dissolution inductively coupled plasma emission spectrometer was used using an inductively coupled plasma emission spectrometer (iCAP7400, Waltham, MA, USA) was used. Available soil N was measured by extracting 2 M KCl and quantified using a continuous flow auto-analyzer (SEAL Analytical GmbH, Norderstedt, Germany) [76]. Soil available P was determined using 0.03 mol L^−1^ of NH_4_F and 0.025 mol L^−1^ of HC1 and then analyzed colorimetrically [77], and available K was analyzed through an inductively coupled plasma atomic emission spectrometer (ICP-AES, IRIS Advantage-ER, Thermo Jarrell Ash Corporation, USA) after digestion in nitric-perchloric acid solution [78,79].

### 4.5. Data Processing and Statistical Analysis

Annual fine root biomass was calculated for each sampling season (March 2018–2019) at each plot by summing up the dry weight of live and dead fine roots in each soil core and scaling up to per hectare. Fine root biomass was calculated for all sampling intervals. We followed the Shapiro-Wilks statistic test for the data normality before the testing trial response. We applied the linear mixed effect model to determine the fine root biomass and nutrient heterogeneity between the monoculture and intercropping systems. Using repeated variables at seasonal intervals, stand ages, cropping systems, and soil depth as fixed factors and plot as a random factor. Post-hoc Tukey tests were performed to examine the difference between the least-square means of each factor. We used the ‘lme4′ and ‘MuMIn’ packages and the “lmer” function to run the model in R. We used the Pearson correlation for fine root biomass and nutrients (N, P, and K). All analysis was performed in R software (Ver.4.1.0) [80].

## 5. Conclusions

The present study elucidated the effect of stand age on the seasonal and vertical distribution (0–60 cm) pattern of fine root biomass and soil available nutrients in rubber monoculture and rubber-*F. macrophylla* intercropping systems. The results showed that stand age, season, soil depth, and cropping systems strongly influenced fine root biomass and soil nutrient availability. Fine root biomass was higher under rubber monoculture and lowered under rubber-*F. macrophylla* intercropping systems. The fine root biomass peaked during the dry season across stand ages, implying that fine roots are positively related to seasons and soil nutrient availability. The nutrient concentration was higher under monoculture plantations to maximize the resource acquisition, which might over yield above-ground biomass in intercropping systems. Further, we provide evidence of stand age effects on soil P and K, which was highest under young plantation (12 years old) than mature plantation (25 years old). P and K peaked in the rainy season, while N peaked in the dry season. Demand for rubber trees on growth and latex production declines with the content of P and K under intercropping systems, particularly P under the mature rubber monoculture. Rubber-*F. macrophylla* intercropping systems will benefit from N-limited soil that significantly reduces the need for N fertilizers. Overall, improving ecological functions will reflect on above-ground and below-ground biomass. Studying and quantifying fine root dynamics are tedious and time-consuming but provide a better understanding of forest productivity.

## Figures and Tables

**Table 1:** Linear mixed-effect model values attained testing effects of different management systems (M), stand age (A), seasons (S), and soil depths (D) on fine root biomass and available nutrients.

Effects		Fine Roots Biomass (Mg·ha^−1^)			Available Nitrogen (mg·kg^−1^)			Available Phosphorus (mg·kg^−1^)			Available Potassium (mg·kg^−1^)		
Factors	*df*	SSq	F Value	*p* Value	SSq	F Value	*p* Value	SSq	F Value	*p* Value	SSq	F Value	*p* Value
Season (S)	4	2.13	5.03	0.001	5.43	70.59	0.001	17.42	12.94	0.001	0.62	5.02	0.001
Management (M)	1	0.25	2.39	1.00	0.00	0.16	0.695	24.14	71.72	0.001	0.93	30.09	0.001
Age (A)	2	0.5	2.38	1.00	0.98	25.38	0.001	114.64	170.27	0.001	0.46	7.49	0.01
Depths (D)	5	191.89	362.47	0.001	28.94	300.82	0.001	95.8	56.92	0.001	7.28	47.22	0.001
S × D	20	4.87	2.3	0.001	--	--	--	--	--	--	--	--	--
A × D	10	2.87	2.71	0.003	--	--	--	17.16	5.1	0.001	--	--	--
S × M	4	0.4	0.95	0.433	0.25	3.29	0.05	13.75	10.21	0.001	0.79	6.38	0.001
S × A	8	1.19	1.4	0.196	1.44	9.34	0.001	35.84	77.82	0.001	0.96	3.9	0.001
M × A	2	0.16	0.77	1.00	0.1	2.6	0.102	52.39	77.82	0.001	0.67	10.84	0.001
M × D	5	0.54	1.03	0.402	--	--	--	1.14	0.67	0.643	--	--	--
S × M × A	8	0.93	1.1	0.362	1.031	6.69	0.001	17.74	6.59	0.001	1.43	5.81	0.001
R^2^	--	--	--	--									
R^2^ m	--	0.76	--	--	0.78	--	--	0.85	--	--	0.64	--	--
R^2^ c	--	0.82	--	--	0.79	--	--	0.87	--	--	0.77	--	--

**Table 2:** Fine roots biomass of different species in the two-cropping systems of different seasonal intervals.

Fine Roots	Age	M.S	S1	S2	S3	S4	S5
Rubber FR	12	Mono	0.52 ± 0.07 a	0.50 ± 0.09 a	0.53 ± 0.08 a	0.53 ± 0.10 a	0.58 ± 0.07 a
		Mix	0.36 ± 0.05 ab	0.37 ± 0.06 ab	0.37 ± 0.05 a	0.42 ± 0.10 a	0.48 ± 0.10 a
	15	Mono	0.34 ± 0.04 ab	0.38 ± 0.06 ab	0.32 ± 0.05 a	0.43 ± 0.10 a	0.45 ± 0.08 a
		Mix	0.33 ± 0.04 ab	0.24 ± 0.03 b	0.37 ± 0.06 a	0.37 ± 0.06 a	0.40 ± 0.08 a
	24	Mono	0.40 ± 0.06 ab	0.47 ± 0.09 ab	0.41 ± 0.09 a	0.43 ± 0.10 a	0.51 ± 0.10 a
		Mix	0.30 ± 0.04 b	0.30 ± 0.05 ab	0.27 ± 0.06 a	0.36 ± 0.09 a	0.32 ± 0.07 a
*F. macrophylla* FR	12	Mix	0.08 ± 0.01 a	0.06 ± 0.01 ab	0.06 ± 0.01 a	0.06 ± 0.01 a	0.09 ± 0.01 a
	15	Mix	0.06 ± 0.01 a	0.12 ± 0.02 a	0.04 ± 0.01 ab	0.05 ± 0.01 ab	0.06 ± 0.01 a
	24	Mix	0.02 ± 0.01 b	0.02 ± 0.01 b	0.02 ± 0.01 bc	0.02 ± 0.01 bc	0.02 ± 0.01 ab
Other FR	12	Mono	0.09 ± 0.02 a	0.04 ± 0.02 a	0.02 ± 0.01 ab	0.01 ± 0.00 ab	0.02 ± 0.01 a
		Mix	0.04 ± 0.01 a	0.00 ± 0.00 a	0.00 ± 0.00 b	0.00 ± 0.00 b	0.00 ± 0.00 a
	15	Mono	0.06 ± 0.02 a	0.05 ± 0.02 a	0.05 ± 0.02 a	0.06 ± 0.03 a	0.04 ± 0.02 a
		Mix	0.01 ± 0.00 a	0.01 ± 0.00 a	0.00 ± 0.00 b	0.00 ± 0.00 b	0.01 ± 0.01 a
	24	Mono	0.01 ± 0.01 a	0.01 ± 0.01 a	0.01 ± 0.01 ab	0.01 ± 0.00 ab	0.01 ± 0.01 a
		Mix	0.00 ± 0.00 a	0.00 ± 0.00 a	0.00 ± 0.00 b	0.00 ± 0.00 b	0.00 ± 0.00 a
Dead FR (Necromass)	12	Mono	0.00 ± 0.00 c	0.01 ± 0.00 a	0.02 ± 0.00 a	0.03 ± 0.01 b	0.01 ± 0.00 a
		Mix	0.01 ± 0.00 c	0.00 ± 0.00 a	0.02 ± 0.00 a	0.12 ± 0.05 a	0.04 ± 0.01 a
	15	Mono	0.15 ± 0.04 bc	0.00 ± 0.00 a	0.03 ± 0.01 a	0.02 ± 0.01 b	0.07 ± 0.02 a
		Mix	0.10 ± 0.02 bc	0.00 ± 0.00 a	0.03 ± 0.01 a	0.09 ± 0.03 b	0.04 ± 0.01 a
	24	Mono	0.35 ± 0.07 a	0.01 ± 0.01 a	0.03 ± 0.01 a	0.05 ± 0.02 b	0.07 ± 0.02 a
		Mix	0.18 ± 0.04 b	0.02 ± 0.01 a	0.01 ± 0.00 a	0.02 ± 0.01 b	0.06 ± 0.02 a

Note: Seasonal intervals (S1, March 2018; S2, June 2018; S3, September 2018; S4, December 2018, and S5, March-19) in the two management systems (M.S), i.e., rubber monoculture (Mono) and rubber-*F. macrophylla* intercropping (Mix). Lower case letters indicate significant differences among different stand ages at each seasonal interval (*p* < 0.05, mean ± SE, n = 3).

**Table 3:** Available nutrients (N, P, and K) concentrations in different seasonal intervals in the two-management systems.

Nutrients	Age	M.S	S1	S2	S3	S4	S5
N (mg·kg^−1^)	12	Mono	80.4 ± 5.1 c	69.4 ± 7.1 bc	68.8 ± 7.3 b	71.2 ± 5.7 c	71.6 ± 5.9 ab
		Mix	82.6 ± 4.6 c	64.0 ± 7.1 c	77.7 ± 7.2 b	58.6 ± 4.7 d	78.0 ± 8.9 a
	15	Mono	101.7 ± 4.3 ab	77.6 ± 7.1 ab	76.3 ± 8.3 b	73.9 ± 7.7 bc	76.4 ± 8.8 a
		Mix	94.4 ± 5.0 b	85.6 ± 9.1 a	91.5 ± 9.3 a	84.7 ± 8.7 a	77.0 ± 7.9 a
	24	Mono	101.3 ± 3.8 ab	80.1 ± 8.1 ab	80.6 ± 9.3 ab	82.2 ± 9.7 ab	72.8 ± 6.9 ab
		Mix	104.5 ± 5.2 a	81.8 ± 7.1 a	76.4 ± 7.3 b	76.1 ± 6.7 abc	62.9 ± 4.9 b
P (mg·kg^−1^)	12	Mono	38.8 ± 4.4 a	50.1 ± 8.3 a	49.5 ± 8.4 a	49.7 ± 4.0 a	41.3 ± 9.4 a
		Mix	2.0 ± 0.2 b	1.4 ± 0.1 b	9.8 ± 2.4 b	1.3 ± 0.9 b	2.5 ± 0.8 b
	15	Mono	1.3 ± 0.1 b	0.7 ± 0.1 b	4.5 ± 1.4 b	1.2 ± 1.0 b	3.4 ± 0.9 b
		Mix	1.6 ± 0.1 b	1.6 ± 0.1 b	1.4 ± 0.6 b	0.7 ± 0.4 b	1.6 ± 0.7 b
	24	Mono	1.4 ± 0.1 b	0.8 ± 0.1 b	0.8 ± 0.4 b	12.3 ± 1.2 b	0.7 ± 0.5 b
		Mix	1.4 ± 0.1 b	0.8 ± 0.1 b	2.7 ± 1.0 b	0.8 ± 0.4 b	1.0 ± 0.5 b
K (mg·kg^−1^)	12	Mono	170.3 ± 9.6 a	211.6 ± 9.2 a	219.7 ± 12.6 a	208.8 ± 14.2 a	188.7 ± 13.9 a
		Mix	86.5 ± 5.8 d	94.3 ± 8.1 bc	94.3 ± 8.8 bc	87.4 ± 9.2 b	102.8 ± 9.2 c
	15	Mono	104.5 ± 6.6 bc	112.8 ± 8.1 b	124.1 ± 10.9 b	116.1 ± 12.1 b	136.5 ± 11.9 b
		Mix	111.2 ± 9.4 bc	88.0 ± 7.2 c	97.7 ± 8.1 bc	97.8 ± 9.8 b	108.4 ± 9.9 bc
	24	Mono	112.8 ± 6.5 b	93.2 ± 7.1 bc	106.6 ± 9.6 bc	113.7 ± 11.2 b	110.3 ± 10.9 bc
		Mix	95.5 ± 9.9 cd	105.5 ± 8.2 bc	87.8 ± 7.6 c	82.0 ± 8.2 b	104.7 ± 9.9 bc

Note. Soil nutrients (N, P, and K) concentration of different ages (12, 15, and 24 years) and seasonal (S) intervals, i.e., S1 (June 2018), S2 (September 2018), S3 (December 2018) and S4 (March-2019) in two management systems (M.S) rubber monoculture (mono) and rubber-*F. macrophylla* intercropping (mix). Data points are mean ± standard error of the two systems; lowercase letters indicate the significant difference in each season in different stand ages. The significance level was determined by a multiple comparisons Tuckey test and is indicated as follows: (*p* < 0.05; mean ± SE, n = 3).

**Table 4:** Basic characteristics of plots.

Age (Years)	M.S	TC (g·kg^−1^)	TN (g·kg^−1^)	TP (g·kg^−1^)	TK (g·kg^−1^)	Soil pH
12	Mono	9.4 ± 1.5 ab	0.3 ± 0.0 a	0.7 ± 0.1 b	20.1 ± 1.6 b	5.8
12	Mix	7.9 ± 1.2 b	1.1 ± 0.1 ab	0.3 ± 0.0 a	17.5 ± 1.0 c	5.9
15	Mono	9.8 ± 0.5 ab	0.3 ± 0.0 a	0.3 ± 0.0 c	16.2 ± 0.9 c	5.0
15	Mix	8.9 ± 0.5 ab	1.2 ± 0.0 a	0.3 ± 0.0 a	15.2 ± 0.6 d	5.0
24	Mono	10.6 ± 0.7 a	1.3 ± 0.1 a	0.3 ± 0.0 c	22.3 ± 3.7 a	5.0
24	Mix	10.8 ± 1.2 a	1.2 ± 0.1 a	0.3 ± 0.0 a	17.3 ± 1.6 c	5.2

Note: M.S; Management system, i.e., rubber monoculture (Mono) and rubber-*F. macrophylla* intercropping (Mix), TC; represents total carbon, TN; total nitrogen, TP; total phosphorus and TK; total potassium. Data points (means ± SE, n = 3) of the two systems; lowercase letters indicate the significant difference in different stand ages.

## Data Availability

Not applicable.
